# Disconnected: What Can We Learn from Individuals with Very Low Nature Connection?

**DOI:** 10.3390/ijerph19138021

**Published:** 2022-06-30

**Authors:** Alexia Barrable, David Booth

**Affiliations:** 1 School of Education and Social Work, University of Dundee, Nethergate, Dundee DD1 4HN, UK; 2 School of Life Sciences, University of Dundee, Nethergate, Dundee DD1 4HN, UK; d.z.booth@dundee.ac.uk

**Keywords:** nature connection, nature disconnection, wellbeing, pro-environmental behaviours

## Abstract

While nature connection, which describes a positive relationship between humans and the rest of the natural world, has been a focus of numerous research studies in the last few decades, relatively little attention has been paid to nature disconnection. While the majority of the populations reported in most studies tend to be highly connected, there is a small percentage of those who feel they have no connection to the natural world. In this paper, we examine this novel construct of nature disconnection through secondary analysis of existing data from the Monitor of Engagement with the Natural Environment survey (MENE) by Natural England. From our analysis of this disconnected population, we can see that they are more likely to be young (16–24 years old), male, not employed and living in rented accommodation. We also observe that they have lower levels of life satisfaction and pro-environmental behaviours. We go on to present an initial theoretical discussion as to the origins of disconnection and propose further research directions to tackle the under-theorisation of this construct.

## 1. Introduction

In the past few decades, researchers in fields such as public health and psychology have increasingly focused on examining the effects that nature has on human health, wellbeing and flourishing. Due to the positive associations of nature contact for human physical and mental health, including diverse types such as animal contact [1], contact with green and blue spaces [2] and even urban nature [3] nature contact has attracted a lot of research interest [4].

At the same time, our relationship to the non-human natural world has been another area of interest. This research has highlighted the many ways humans are connected to non-human nature and our deep interdependence with our environment [5]. Drawing from work such as the *biophilia hypothesis* [6], which proposes that humans have an innate need to feel close and affiliate with the rest of the natural world, environmental psychology and other related fields have examined our relationship to the natural world.

### 1.1. Nature Connection

Nature connection, which describes a positive relationship between humans to the rest of the natural world, has been seen an increase in research interest in environmental psychology [7,8] and other related fields, such as education [9] and mental health research [10].

One of the driving factors has been previous research that has found positive associations between nature connection and increased wellbeing, including eudaimonic wellbeing [11], life satisfaction [12], vitality [13] and positive affect [14]. Meta-analyses have found that these correlations range from r = 0.17 for life satisfaction [12] to r = 0.24 for eudaimonic wellbeing [11] and vitality [12].

On the other hand, recent meta-analyses have shown that nature connection is significantly and positively associated with pro-environmental attitudes and behaviours [15]. Mackay and Smith [15] found a moderate to high association between pro-environmental behaviours and nature connections. Whitburn et al. [16] separated correlational and experimental studies in their meta-analysis, reporting a bigger effect for correlational data and a smaller effect in experimental studies. Given the current climate and biodiversity crises, nature connection has been seen as a way to increase drive and motivation to act in pro-environmental ways.

As such, nature connection has become a focus of different types of research, including experimental research that looks at interventions to enhance nature connection, both in children [17] and in adults [18,19,20]. Moreover, we have also seen the cross-sectional measurement of nature connection in more extensive populations, childhood and adolescence [21,22] and in adulthood [23]. These previous studies have seen higher levels of nature connection in younger populations, with a dip in adolescence. Moreover, interventions in younger children below the age of nine tend to be more effective than those in older children and adults. More longitudinal work is needed to explore the longevity of the effects in all populations.

Nature connection has many related constructs and measures [24], such as Inclusion of Nature in Self [25], Nature Relatedness [26], and Connection to Nature [27]. These measure various dimensions and aspects of a positive human-nature relationship and include trait, meaning a fairly stable personality characteristic, or state, a more ephemeral and changing quality. Given the aforementioned increasing importance of the role of nature connection in promoting pro-environmental behaviours and positive mental health, we have now seen a nature connection measure, the Nature Connection Index (NCI) [28], included in an existing national survey taking place in England, the Monitor of Engagement with the Natural Environment Survey (MENE).

Contrary to the popular narrative of ‘disconnection from nature’ [29] and nature-deficit disorder in children [30], most of the time, when measuring nature connection in a population, there is a distinct negative skew in the distribution, with the majority of the population being at the high end of the spectrum and with a long tail at the low end [28]. This is linked to an oft-seen ceiling effect that has been observed, especially in interventions [17]. Although this is potentially a side-effect of the measurement instruments used, as Richardson et al., 2019 [28] suggest, and newer instruments, such as the NCI, have a more normal distribution, this paper aims to bring attention to and examine the not-previously-studied population that is at the lower end of the spectrum in relation to nature connection: A disconnected population.

### 1.2. Nature Disconnection

Ulrich [31], in the seminal book on the Biophilia Hypothesis [6], proposes the term biophobia to describe an aversion to certain natural elements such as snakes. The two terms, biophilia and biophobia, are respectively related to positive, or approach, responses for the former and negative, or aversive, responses for the latter. Moreover, in a rare study of nature aversion, described as ‘discomfort or awkwardness in a natural environment’ (p. 161), Lee & Lee [32] found that it is correlated with limited nature experiences and a low frequency of encounters.

While nature connection has been studied extensively in the last twenty years, we can see from large population surveys that there is a part of the population that is at the very low end of the spectrum. In this paper, we propose the need for a distinct construct to describe this state of very low nature connection, henceforth referred to as ‘nature disconnection’. In the first part of the paper, we use a ‘research-question driven’ secondary data analysis [33] to explore the demographic and other characteristics of this sub-set of the population, as well as the associations of low nature connection with wellbeing and pro-environmental behaviours, in line with previous research on nature connection that has seen robust associations, as presented above. We then go on to examine possible conceptualisations of nature disconnection, including potential dimensions and then we explore avenues for further research. These three research questions will be put forward:

Research Question 1: What are the demographic and other characteristics of nature disconnected people?

Research Question 2: How does nature disconnection associate with wellbeing (physical and psychological)?

Research Question 3: How does nature disconnection associate with pro-environmental behaviours?

## 2. Method

### 2.1. Participants

The data we used for this secondary data analysis came from the Department for Environment, Food and Rural Affairs commissioned a survey to monitor engagement with the natural environment (MENE) [34]. These data were collected face-to-face at home by interviewers in the form of computer-assisted interviews of omnibus surveys conducted throughout England between 2009 and 2019, covering a wide range of related topics. The year 1–10 visit data for adults were chosen as an initial testbed for exploring disconnected individuals. A subset of this data that was complete for variables of interest was used (*n* = 4735, 2450 female, 16–65+ years of age).

### 2.2. Measures

Nature connection was measured using the NCI [28], which was developed as a short and reliable measure of nature connection to be used in a variety of contexts, including, in this case, a nationwide survey. The NCI is a six-item, seven-point Likert scale (1 = “completely disagree” to 7 = “completely agree”) response. It is a unidimensional measure that aims to capture the person’s relationship with the rest of the natural world (example items include “Being in nature makes me very happy.”). The scoring is weighted [35] and results in scores from 0 to 100. The measure is seen as a valid and reliable measure of nature connection, and more information on its development and psychometric characteristics can be seen in Richardson et al. [28].

Psychological wellbeing was measured on a one-question, eleven-point scale response, with answers ranging from 0 through to 10, including ‘don’t know’ to the question ‘Overall, how satisfied are you with life nowadays?’, as per the Office of National Statistics surveys [35]. We also used the general health question to operationalise physical health, which was measured with a self-reported health question with five categories ranging from ‘very good’ through to ‘very bad’ in addition to ‘don’t know’.

Finally, pro-environmental behaviours were given in a nine-question, binary ‘yes’ or ‘no’ response to questions such as ‘I usually recycle items rather than throw them away’ and ‘I usually buy eco-friendly products and brands’. Answers to all the questions were aggregated to compute a general PEB factor with a score ranging from 0 to 10.

### 2.3. Defining Nature Disconnection

**Figure 1:**
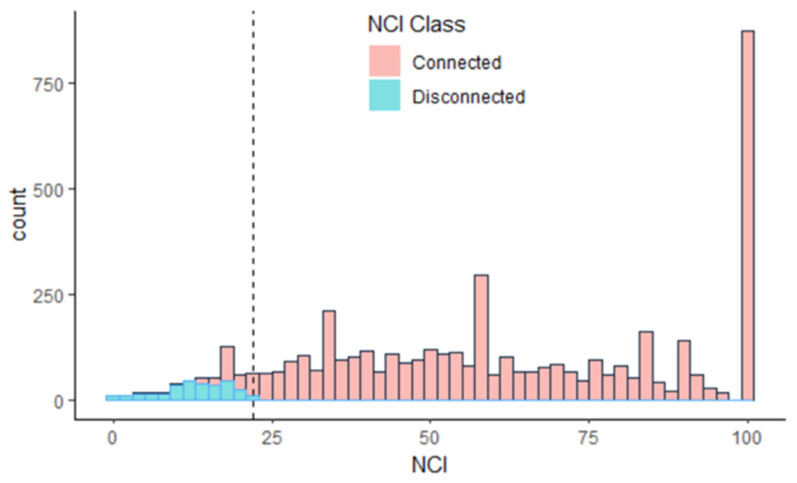
Distribution of NCI scores of adult MENE respondents, classified as connected and disconnected, dashed line indicates an NCI = 22 with blue bars indicating those responding negatively to statement six: “I feel part of nature”.

To undertake the research-question-driven secondary data analysis, we initially had to decide how to define nature disconnection. Overall NCI scores (*M* = 60.38, *SD* = 28.18) had two notable properties, a strong negative skew and large proportion (0.18) of individuals at the ceiling of the scale. Likewise, to the NCI scale question, “I feel part of nature”, a large proportion responded either neutrally or positively (0.85). As such disconnected individuals were identified using the following two criteria: they had to both be in the lowest decile of overall scores, corresponding to an NCI equal to or lower than 22, as well as having expressed a negative response to “I feel part of nature”. This split the population into connected (*N* = 4421, 94%), and disconnected (*N* = 314, 6%), see Figure 1. Here we report on the 6% of the population at the low end of the scale. 

### 2.4. Predictors

Demographic data included the categorical variables of sex (female, male) and age (six categories from 16–24 through to 65+). Marital status was classed into married, single, and separated/divorced/widowed. The socioeconomic group were collected and classed based on occupation as per the UK social grade ranging from A (i.e., professionals) through to E (Non-working), complemented by working status, which is a five-category variable ranging from full-time through to retired. Tenure was the legal status under which people had the right to occupy accommodation, owning outright, mortgage, renting private, and from local authorities and other. Respondents’ residential areas were ranked in an index of mean deprivation (IMD). These were divided into quartiles with the following IMD values (0.00 = 33, 0.25 = 6379, 0.50 = 12,986, 0.75 = 21,623, 1.00 = 32,814) from the least deprived to the most deprived.

### 2.5. Analysis

All graphing and analyses were conducted in the R environment [36]. Predictors were correlated with the binary classification using a point-biserial Pearson model for binary variables (age, sex), Kendall’s *τ* for ordinal (Socioeconomic group, Mean Deprivation, Wellbeing, Pro-environmental behaviour sum) and *χ*^2^ for nominal (General Health, Marital status, Working status and Tenure). For *χ*^2^ effect size, *φ* was calculated.

A generalised linear model (GLM) with binomial error and logit link function was fitted to the binary classified respondents, where 0 corresponded to those considered ‘connected’ and 1 ‘disconnected’. The following set of terms, with no interactions, were fitted to produce the following saturated GLM. Multicollinearity was estimated using a generalised variance inflation factor approach for categorical predictors [37].

Disconnected ~ sex + age + marital status + working status + socioeconomic group + tenure + mean deprivation + wellbeing + general health + pro environmental behaviours summed

A likelihood ratio test [38] was performed to contrast the model with a null model with no predictors. To conservatively control for false positives, the Benjamini and Hochberg [39] approach with a (false discovery rate = 0.25) was applied to the model *p*-values. Odds ratios and confidence intervals were calculated as the exponential of the predictor; the regression model is presented as a Supplementary Table S1 and Figure S1.

## 3. Results

### 3.1. Research Question 1: Demographic Characteristics

Correlation coefficients between various demographics and nature disconnection are presented in Table 1. Negative values indicate a negative association, i.e., a reduction in disconnection as the variable increases, while positive values indicate a positive association, i.e., higher nature disconnection as the variable increases. As such, younger ages, in this instance, the 16–24 age group, were observed to be more highly disconnected than others, with an increase in age category reducing the likelihood of disconnect; for example, ages 35–44 significantly differed from the reference *B* = −0.66, *SE* = 0.22, *p* = 0.025, with the odds ratio favouring a decrease in disconnect [*OR* = 0.51, 95% CI (0.31, 0.83)]. This trend continues through all age classes until 65+ at which point disconnect is equivalent to the 16–24 age group. Sex was found to be significantly correlated with nature disconnection differing from females *B* = −0.29, *SE* = 0.12, *p* = 0.007, with the odds ratio favouring an increase in disconnect in males [*OR* = 1.34, 95% CI (1.04, 1.73)]. Marital status correlated with disconnect, with this association driven by the difference between married and single individuals *B* = −0.31, *SE* = 0.16, *p* = 0.046, with the odds ratio favouring an increase in a disconnect in single individuals [*OR* = 1.36, 95% CI (1.00, 1.85)]. Whilst nominal employment and lower socioeconomic status (SES) do correlate with the disconnect; the regression model found no significant predictors within those variables. In relation to tenure, those renting from a local authority were more disconnected than those owning outright *B* = −0.37, *SE* = 0.20, *p* = 0.056, with the odds ratio favouring an increase in disconnect in those renting from a local authority [*OR* = 1.46, 95% CI (0.99, 2.17)]. 

### 3.2. Research Question 2: Wellbeing (Psychological and Physical)

For the second research question, we looked at associations of psychological and physical wellbeing with nature disconnection, as defined previously. Psychological wellbeing, in the form of the single question on life satisfaction, was found to be significantly and negatively correlated with nature disconnection, with a higher level of disconnection associated with higher dissatisfaction with their lives (*τ* = −0.06, *p* < 0.001). This association per unit increase in wellbeing *B* = −0.10, *SE* = 0.03, *p* < 0.001, with the odds ratio favouring a decrease in disconnect as wellbeing increases [*OR* = 0.91, 95% CI (0.86, 0.96)]. On the other hand, self-reported physical health, in this instance, operationalised as physical wellbeing, was not found to be significantly correlated with nature disconnection (*φ* = 0.05, *p* = < 0.080).

### 3.3. Research Question 3: Pro-Environmental Behaviours and Nature Disconnection

The third and final question we had set to investigate was whether nature disconnection was associated with pro-environmental behaviours. In this instance, we observed that PEBs, as measured by an aggregated set of questions, were negatively correlated with nature disconnection (*r* = −0.18, *p* < 0.001). In fact, PEB seemed to be the factor most correlated with nature disconnection, and per unit increase in score *B* = −0.59, *SE = 0*.05, *p* < 0.001, with the odds ratio favouring a decrease in disconnect as PEB increased [*OR* = 0.55, 95% CI (0.49, 0.61)].

## 4. Discussion

In this paper, we introduced the little-studied construct of nature disconnection and used existing data to explore the demographic characteristics of those who score at the lowest end of the NCI. We also wanted to see if the correlations found to hold true in the general population, in previous research, including several meta-analyses, namely with wellbeing and PEBs were present in this disconnected population.

Findings suggest that young (16–24) single males not employed and not owning a house are more likely to be disconnected from nature, with each additional factor further heightening the risk of being disconnected. Furthermore, we found that disconnected people are more likely to be dissatisfied with their lives but not more likely to suffer more ill health, as per the self-reported measure used in the survey. The former finding agrees with previous literature [11,12] and the measures are comparable in size. Finally, people who in this paper have been identified as disconnected from the natural world seem to be less inclined to undertake pro-environmental behaviours, such as recycling or buying local and seasonal produce. Again this finding concurs with previous meta-analyses on this topic [15,16].

More importantly, the conceptual point we wish to put forward in this paper is that being disconnected is potentially not simply the lack of connection, just as negative affect is not the absence of positive affect, although this may well be one of the characteristics. More than that, disconnection goes against the prevalent characteristics of the general population. We would like to argue that if we take nature connection as stemming from an innate affinity with the natural world, as per the biophilia hypothesis, then being disconnected is somehow ‘abnormal’. Disconnection is not simply a product of a lack of positive experiences, given the innate nature of biophilia. Arguably, nature disconnection may be a product of disordered experience: negative or fearful, uncomfortable or aversive encounters. Another possibility would be a disruption in the development of a positive relationship with the natural world in childhood, given that most of the nature connection literature recognised childhood as a critical period for developing a positive human-nature relationship [7,17]. Alternatively, and the above data may hint at an indication of this, it may be a cultural artefact or a complex combination of factors. As such, it may be an artefact of specific cultural conditions, such as unemployment or another disenfranchisement, that brings disconnection and separation not just from the natural world but potentially from the rest of society too. We wish to highlight that these hypotheses need to be further studied rather than linked to the data presented in this paper.

To further add to the theorisation of this understudied population, we return to Metzner [40] and his writings on the psychopathology of the human-nature relationship. Metzner relies on diagnostic metaphors to describe the unnatural split between humans and her environments, such as amnesia and dissociation. Moreover, he highlights the pathological aspects of a negative relationship and our ‘destructive or exploitative treatment of the natural world’, ([40], p. 56). He does not, however, attribute this broken relationship solely to the individual but includes within his explanation societal and other influences. As the study of nature disconnection grows, such exploration of the role of individual vs. societal influences will need to be explored. 

### 4.1. Limitations

This paper represents the first foray into the construct of nature disconnection and does so using existing data from the MENE. As such, and as is often the case when using secondary data, our questions were limited by the data that were collected at the time [33]. As such, we could not explore questions that would have given us more information on some of the characteristics of the subsample analysed. Similarly, as these variables are collected concurrently and without experimentation, causality cannot be inferred, only association.

Furthermore, within those data, the measures that were used were those suited to omnibus surveys, which in the case of some of the measures, e.g., wellbeing, did not allow for much granularity. Given this limitation, it is still of interest that significant associations were observed. Additionally, these associations were comparable to previous research results.

A notable limitation of this type of secondary analysis is that it can easily miss confounding variables that may not have been included in the initial dataset. Moreover, it is important here to acknowledge the limitations of quantitative research in explaining or delving deeply into the phenomenon in question, nature disconnection. As such, we suggest that further research should be conducted.

### 4.2. Further Research

One of the first steps in the process of more deeply understanding the characteristics of nature disconnection at the population level, and therefore using quantitative research, would be the development of a scale or measure. While a wide variety of psychometric scales exist to measure nature connection [24] at the moment, and as a side-effect of the little attention that has been paid to the construct itself, no scale exists for nature *dis*connection. Such a measure would be useful in large-scale correlational, longitudinal and experimental studies in future. Moreover, it would help us understand the impact that nature-based interventions, often used to support health and wellbeing [3], may have on such populations, who start off as disconnected.

One of the questions that inevitably gets posed when a new construct is proposed, as in this instance, and especially when considering the development of a measure, is its dimensionality. Despite ongoing debates as to the dimensions of nature connection, multi-dimensional measures, such as the Nature Relatedness Scale (NR) [13] and the CN-12 [41] tend to have better performance, in psychometric terms [24]. As such, we can theorise that nature disconnection is very likely to also be a multi-dimensional construct, potentially including separation or alienation, aversion, and experiential dimension. These are proposed in line with similar nature connection measures with dimensions of oneness or inclusion of self, aesthetic appeal/beauty, and experiential dimensions of physical interaction with nature. We also propose an extra dimension for nature disconnection, coming from biophilia literature, and that is fear (biophobia).

Another important next step for future research would be qualitative studies that explore the mindset of people who experience nature disconnection, as well as their experience of nature. The latter should be looked at both through an exploration of past negative experiences that may have precipitated aversive or fearful feelings and lack of experience in natural spaces and at a perceptual level. Only through a pragmatic approach and the deployment of mixed methods research [42] can we truly explore and explain the complex relationship (or separation) between humans and nature.

## 5. Conclusions

Nature connection, the construct that describes an individual’s positive relationship with the natural world, has increasingly been the focus of research in environmental psychology and cognate fields. Its antithetical partner, nature disconnection—a newly coined and proposed construct in this paper—has attracted little attention. In this paper, we looked at some of the demographics correlated with nature disconnection in a large sample (*n* = 4735) of English residents in an attempt to understand the profile of a disconnected person. As such, we see that disconnected people are more likely to be young, single males. Moreover, we identified two variables of interest, namely wellbeing and PEBs, which have previously been found to be positively linked with nature connection, and explored how, if at all, they are associated with nature disconnection. Analysis suggested a similar but potentially smaller association.

This paper makes a start in the conceptualisation of nature’s disconnection and provides an initial approach to its theorisation. Further work should be directed towards constructing a psychometric scale that can be used in research to further explore associations with behaviours, previous experiences, personality characteristics and psychological states, but also to be used diagnostically to pinpoint how and why nature disconnection occurs.

## Figures and Tables

**Table 1:** Correlation coefficients for demographic characteristics against nature disconnection.

	Correlation Coefficient	*p*
Age	−0.07	<0.001
Sex	0.05	<0.001
Marital Status	0.10	<0.001
Working Status	0.07	<0.001
SES	0.04	<0.001
Tenure	0.08	<0.001
Mean deprivation index	0.08	<0.001

## Data Availability

Data available at http://publications.naturalengland.org.uk/publication/4897139222380544 (accessed on 24 May 2022).

## Supplementary Materials

The following supporting information can be downloaded at: https://www.mdpi.com/article/10.3390/ijerph19138021/s1, Figure S1: Forest plot of odds ratios from GLM of predictors of disconnection; Table S1: Logistic regression model predicting nature disconnect as a function of demographics and social characteristics/behaviours from MENE dataset.
