# Supplementary material for: Disconnected: What Can We Learn from Individuals with Very Low Nature Connection?
Source: Int J Environ Res Public Health. 2022 Jun 30;19(13):8021. doi: 10.3390/ijerph19138021 (PMC9266168; doi:10.3390/ijerph19138021)
Supplement: Supplementary file 1 [file ijerph-19-08021-s001.zip › ijerph-1764468-supplementary.pdf]

Supplementary Materials

Overall regression table

Table S1. Logistic regression model predicting nature disconnect as a function of demographics and social characteristics/behaviours from MENE dataset. Odd's ratios indicate odds of being disconnected. N = 4735, $\chi2 = 291.46$, $p < 0.001$, McFadden's pseudo $R^2 = 0.14$. Multicollinearity was deemed to be low as using the generalized variance-inflation factor approach for categorical predictors $GVIF^{(1/(2*df))}$, which when squared produced values ranging from 1.07–1.94.

	Nature disconnect				
	**Beta**	**OR**	**95% CI OR**	**z value**	***p***
**Age**					
16-24 (reference)	-	-	- -	-	-
25-34	-0.363	0.696	[0.454 - 1.068]	-1.666	0.096
35-44	-0.669	0.512	[0.312 - 0.834]	-2.675	0.007
45-54	-0.637	0.529	[0.320 - 0.868]	-2.509	0.012
55-64	-0.782	0.457	[0.260 - 0.791]	-2.761	0.006
65+	-0.683	0.505	[0.233 - 1.087]	-1.739	0.082
**Sex**					
Sex (Male)	0.291	1.337	[1.039 - 1.725]	2.247	0.025
**Marital Status**					
Married (reference)	-	-	- -	-	-
Sep/Wid/div	-0.097	0.908	[0.602 - 1.349]	-0.472	0.637
Single	0.310	1.363	[1.004 - 1.846]	1.994	0.046
**Working status**					
Full time (reference)	-	-	- -	-	-
In Education	0.218	1.244	[0.745 - 2.061]	0.843	0.399
Not working	0.107	1.113	[0.742 - 1.656]	0.523	0.601
Part time	-0.034	0.966	[0.619 - 1.477]	-0.156	0.876
Retired	0.325	1.384	[0.687 - 2.778]	0.910	0.363
**Socioeconomic group**					
A (Reference)	-	-	- -	-	-
B	-0.324	0.723	[0.318 - 1.872]	-0.727	0.467
C1	-0.597	0.551	[0.250 - 1.397]	-1.378	0.168
C2	-0.783	0.457	[0.205 - 1.167]	-1.789	0.074
D	-0.632	0.532	[0.237 - 1.362]	-1.437	0.151
E	-0.581	0.560	[0.246 - 1.451]	-1.299	0.194
**Tenure**					
Owned outright (reference)	-	-	- -	-	-
Mortgage	0.433	1.543	[1.001 - 2.380]	1.597	0.110
Rent private	0.170	1.186	[0.783 - 1.801]	-0.156	0.876
Rent local authority	0.372	1.451	[0.984 - 2.149]	2.979	0.003
Other	0.076	1.079	[0.501 - 2.148]	0.809	0.419
**Mean Deprivation**					
First quartile - least deprived (reference)	-	-	- -	-	-
Second quartile	0.245	1.277	[0.853 1.931]	1.177	0.239
Third quartile	0.321	1.379	[0.925 2.080]	1.557	0.120
Forth quartile - most deprived	0.479	1.615	[1.083 2.441]	2.317	0.021
**Wellbeing**					
Overall life satisfaction	-0.098	0.906	[0.857 - 0.959]	-3.461	0.001
**Pro-environmental behaviours**					
Qe4 sum	-0.589	0.555	[0.498 - 0.616]	-10.876	<0.001
(Intercept)	-0.713	0.490	[0.156 - 1.405]	-1.283	0.200

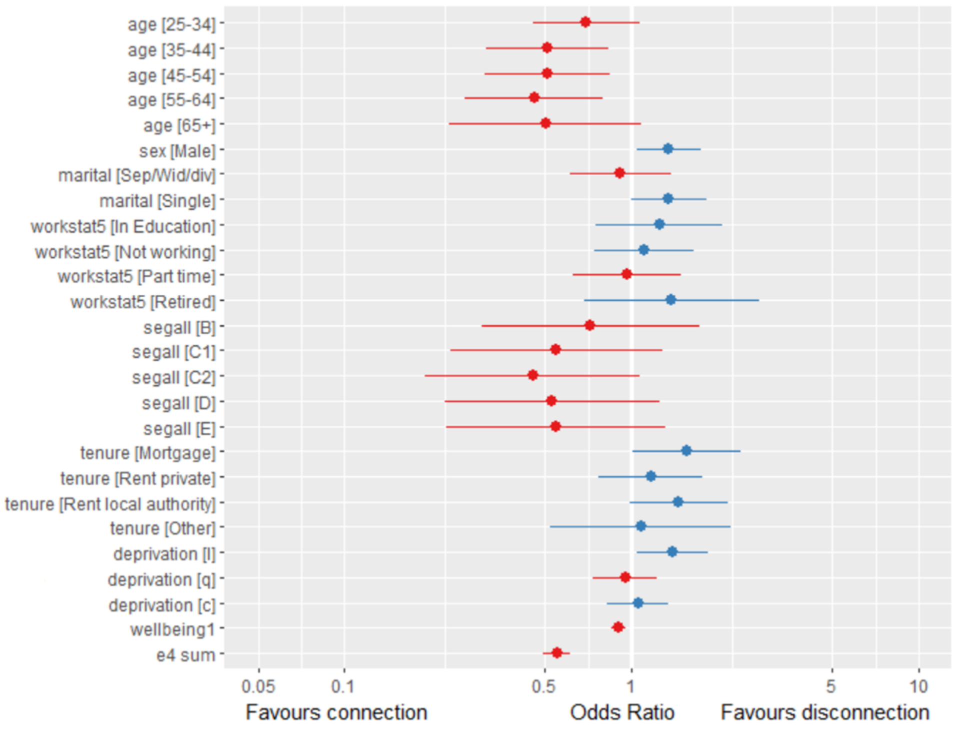

Figure S1. Forest plot of odds ratios from GLM of predictors of disconnection. Values to the right of the centre line (odd's ratio of 1) indicate that disconnection is favoured, values to the left indicate connection is favoured.
